# Flexible Bronchoscopic Retrieval of an Aspirated Tooth in Severe Radiation-Induced Trismus: A Case Report

**DOI:** 10.7759/cureus.115162

**Published:** 2026-08-25

**Authors:** Dainamar Perez-Gonzalez, Juan Irizarry-Nieves, William Rodriguez-Cintron, Yomayra Otero-Dominguez, Edgardo Adorno-Fontanez

**Affiliations:** 1 Pulmonary and Critical Care Medicine, VA Caribbean Healthcare System, San Juan, PRI

**Keywords:** aspirated tooth, flexible bronchoscopy, foreign body aspiration, head and neck cancer, radiation-induced trismus, trismus

## Abstract

Foreign body aspiration is uncommon in adults but can result in significant morbidity, particularly among head and neck cancer survivors, who frequently develop dysphagia, impaired airway protection, and radiation-induced trismus.

We report the case of an 82-year-old man with a remote history of head and neck squamous cell carcinoma treated with radical neck dissection and radiotherapy who aspirated an extracted molar during a dental procedure complicated by severe trismus. Computed tomography demonstrated the tooth within the right bronchus intermedius. Rigid bronchoscopy was considered unsafe because of restricted mouth opening, and standard bite-block placement was not feasible. Flexible bronchoscopy was therefore performed through a nasopharyngeal approach under moderate sedation. The tooth was captured with a retrieval basket and withdrawn into the oropharynx. Because the irregular dental fragment posed a risk of mucosal injury during extraction through the restricted oral aperture, the patient was repositioned in a slight Trendelenburg and left lateral decubitus position, allowing for successful manual removal with forceps.

This case highlights the technical challenges of foreign body retrieval in patients with radiation-induced trismus. In this patient, a staged transnasal flexible bronchoscopic approach followed by direct manual extraction allowed successful foreign body retrieval when conventional rigid or oral bronchoscopic techniques were not feasible. This approach may represent a feasible alternative in carefully selected patients with similar anatomic constraints.

## Introduction

Foreign body aspiration in adults is uncommon but may result in significant morbidity, particularly in elderly patients and those with conditions that impair swallowing or airway protection [1]. In a multicenter study of 138 adults with tracheobronchial foreign body aspiration, the mean age was 66.3 years, and teeth were the most frequently identified foreign body (37.7%), followed by chicken bones (15.2%), nuts (14.5%), and fish bones (9.4%) [1]. Iatrogenic events accounted for 37.0% of cases [1]. Trismus is also a recognized complication among patients treated for head and neck cancer and may significantly restrict mouth opening [2].

Head and neck cancer survivors treated with radiotherapy represent a particularly vulnerable population because radiation therapy can result in dysphagia, sensory impairment, xerostomia, dental deterioration, and trismus, all of which increase the risk of aspiration and complicate airway management [3,4]. Radiation-induced trismus is a recognized late complication that may substantially restrict mouth opening, limiting access for diagnostic and therapeutic airway procedures [4].

Rigid bronchoscopy remains the standard approach for removing large or irregular tracheobronchial foreign bodies because it provides excellent airway control and accommodates larger retrieval instruments [5]. However, severe trismus may preclude safe insertion of a rigid bronchoscope or even standard bite blocks required for oral flexible bronchoscopy [6]. In these situations, flexible bronchoscopy has emerged as an effective alternative, with reported retrieval success rates approaching 90% in adults [1,7].

We report the successful retrieval of an aspirated molar in an 82-year-old man with severe radiation-induced trismus using a staged approach consisting of transnasal flexible bronchoscopy followed by direct oropharyngeal extraction with forceps under optimized patient positioning. This case highlights a practical alternative for airway foreign body retrieval when conventional rigid or oral bronchoscopic approaches are not feasible.

## Case presentation

An 82-year-old man with a history of moderate persistent asthma, chronic sinusitis, and squamous cell carcinoma of the head and neck treated with radical neck dissection and radiotherapy to the right temple and parotid gland in 1999 presented for elective extraction of carious teeth #30 and #32. Preprocedural dental evaluation demonstrated a maximum interincisal opening of approximately 15mm-20 mm, measured using a millimeter ruler, consistent with severe radiation-induced trismus. Despite the restricted oral aperture, extraction was attempted, during which tooth #32 became dislodged and was aspirated.

**Figure 1:**
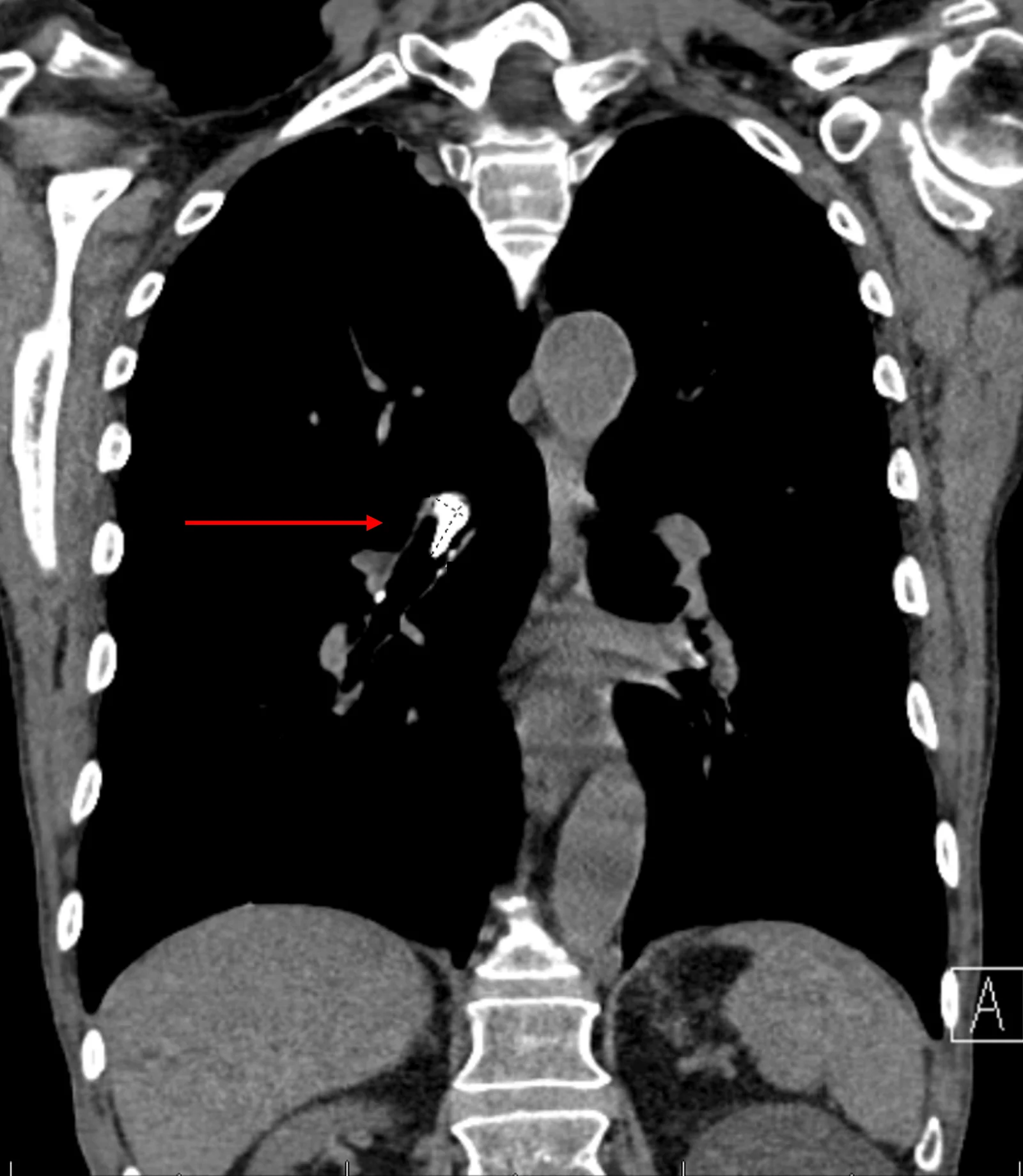
Contrast-enhanced coronal CT of the chest demonstrating a radiopaque aspirated molar (red arrow) lodged within the proximal right bronchus intermedius CT: computed tomography

Immediately following the event, the patient was breathing comfortably, with oxygen saturation of 97%-99% on room air and no abnormal breath sounds on auscultation. He subsequently reported a nonproductive cough but denied dyspnea, chest pain, or hemoptysis. Contrast-enhanced computed tomography (CT) of the chest, obtained approximately five hours after the dental procedure, demonstrated a radiopaque foreign body lodged within the right bronchus intermedius (Figure 1).

On pulmonary evaluation, the patient remained afebrile and hemodynamically stable, with a blood pressure of 110/54 mmHg, heart rate of 73 beats/min, respiratory rate of 17 breaths/min, temperature of 98.1°F (36.7°C), and oxygen saturation of 96%. He was in no respiratory distress, and pulmonary examination demonstrated well-ventilated lungs that were clear to auscultation without focal wheezing or diminished breath sounds. Laboratory studies showed no clinically significant abnormalities. Bronchoscopic retrieval was planned within 24 hours of the aspiration event.

Preprocedural airway evaluation revealed a Mallampati class III airway with preserved neck extension. Given the patient’s markedly restricted oral opening, rigid bronchoscopy was considered but deemed technically unsafe because the rigid bronchoscope and standard adult bite block could not be accommodated. Pediatric bite blocks were unavailable at our institution. After multidisciplinary discussion, flexible bronchoscopy via a transnasal approach under moderate sedation was selected to preserve spontaneous ventilation and maintain an intact cough reflex should the foreign body become dislodged during retrieval.

**Figure 2:**
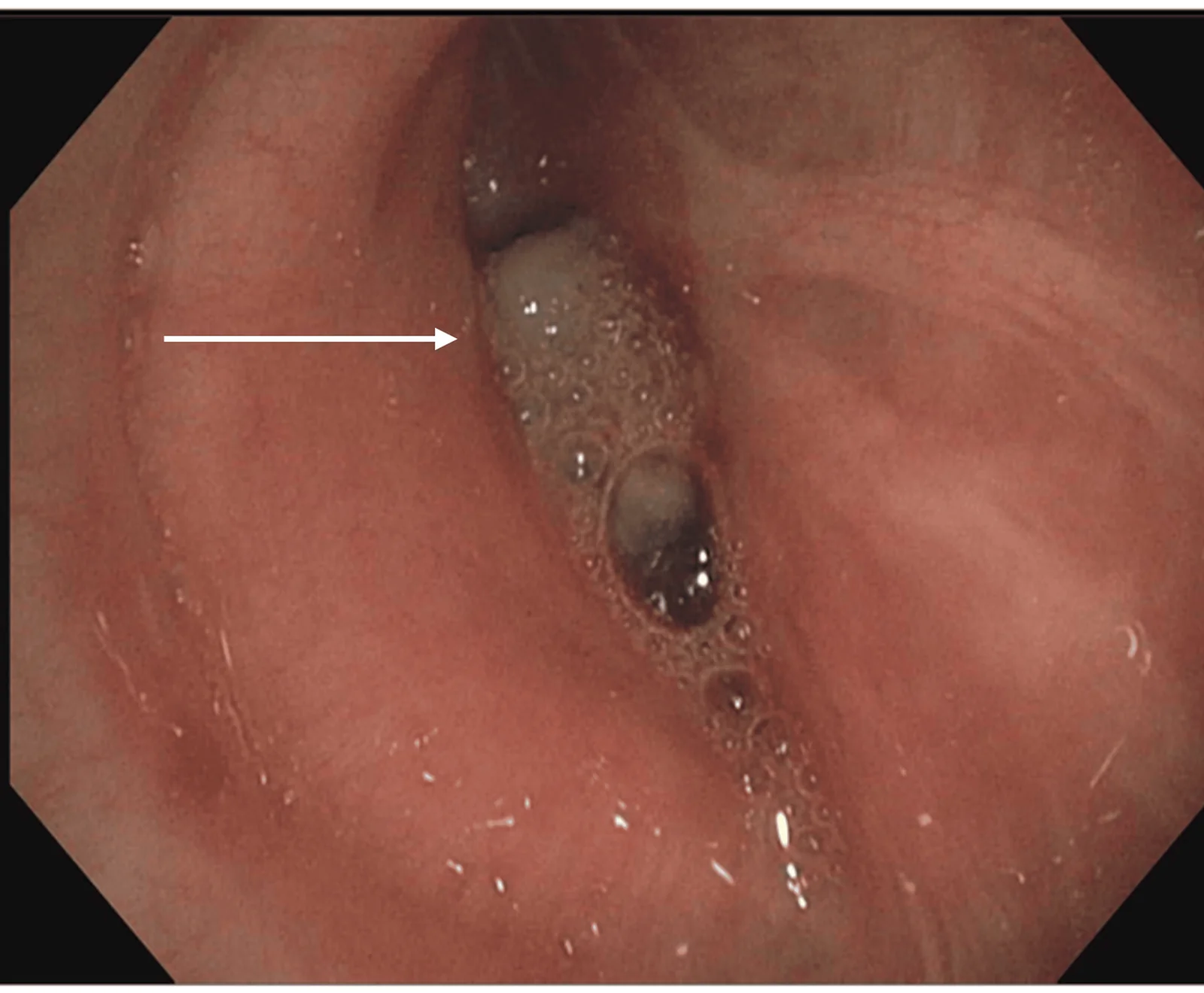
Flexible bronchoscopic view of the aspirated molar (white arrow) lodged within the right bronchus intermedius distal to the secondary carina, obtained via a transnasal approach

Topical anesthesia consisted of 2% lidocaine gel applied to the nasal passages and 1% lidocaine administered to the epiglottis and tracheobronchial tree. Moderate sedation was achieved with intravenous midazolam and fentanyl. A BF-Q190 flexible bronchoscope (Olympus Medical Systems, Tokyo, Japan) with a 4.8-mm outer diameter and a 2.0-mm working channel was introduced through the right naris. The vocal cords were traversed without difficulty, and bronchoscopy identified the aspirated molar lodged in the proximal right bronchus intermedius, just distal to the secondary carina (Figure 2).

**Figure 3:**
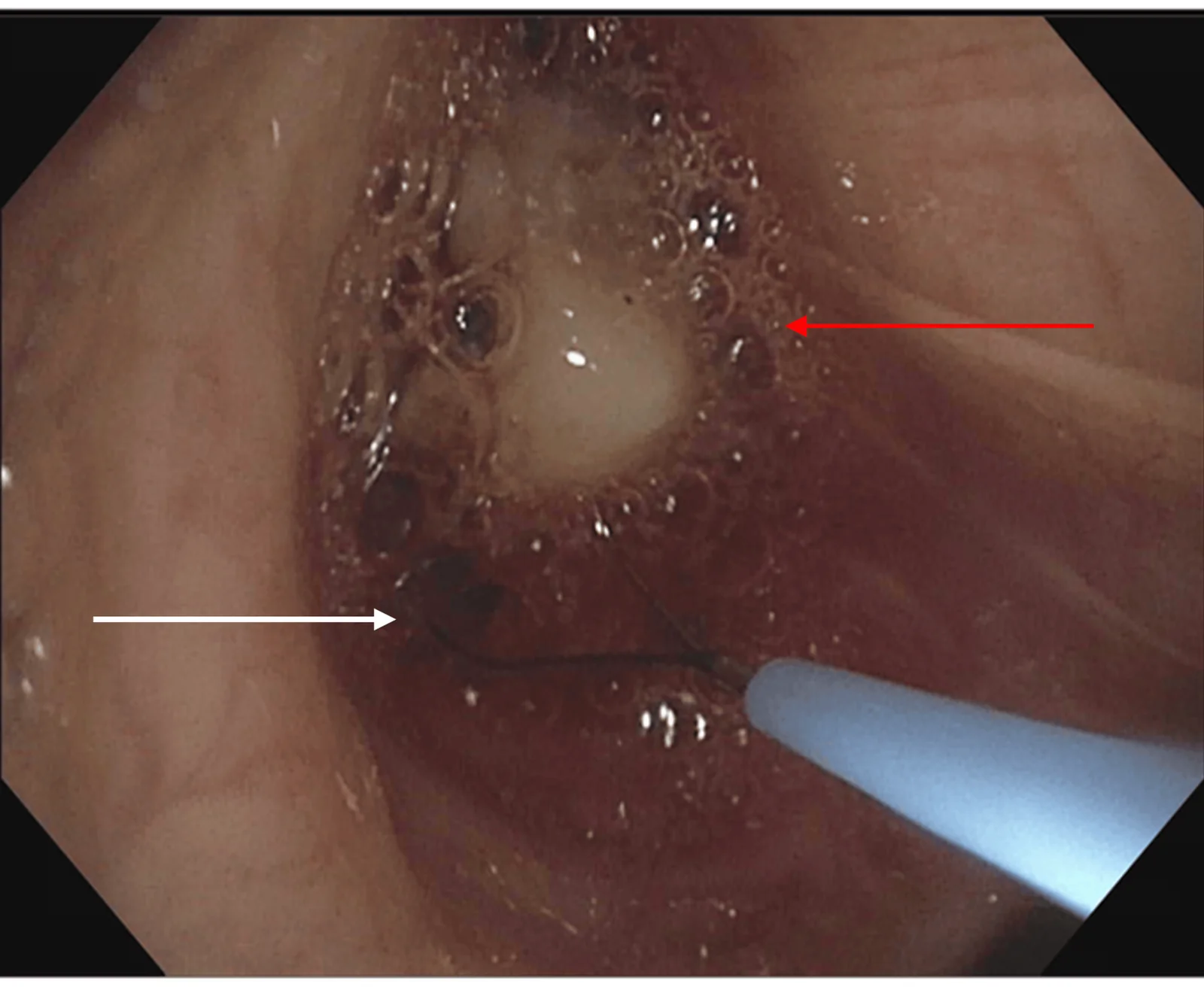
Flexible bronchoscopic view demonstrating the retrieval basket (white arrow) engaging the aspirated molar (red arrow)

**Figure 4:**
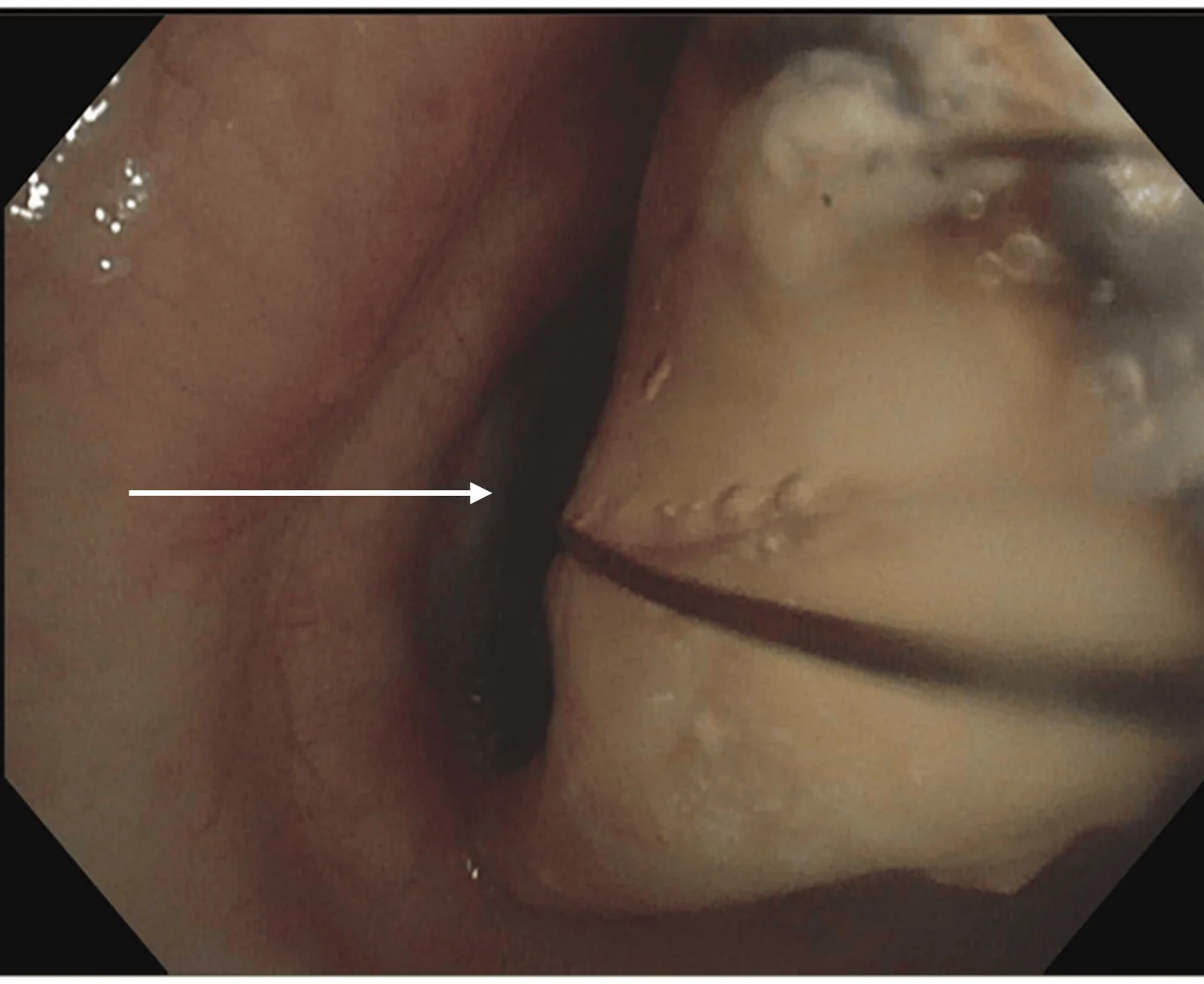
Flexible bronchoscopic view demonstrating the aspirated molar securely captured within the retrieval basket (white arrow)

A retrieval basket was advanced through the working channel beyond the foreign body, deployed distal to the tooth, and withdrawn proximally to securely capture it (Figure 3 and Figure 4).

The bronchoscope, retrieval basket, and entrapped tooth were then withdrawn together through the vocal cords into the oropharynx. However, complete extraction through the severely restricted oral aperture was considered unsafe because the irregular contour and sharp enamel edges of the tooth posed a risk of injury to the soft palate, posterior pharyngeal wall, and bronchoscope.

To minimize the risk of re-aspiration during transfer of the foreign body, the patient was repositioned in a slight Trendelenburg and left lateral decubitus position to facilitate gravity-assisted movement toward the oropharynx. Continuous suction was maintained by an assistant at the bedside during the oropharyngeal extraction. Standard Kelly forceps were introduced through the restricted oral aperture, and the tooth was directly grasped in the oropharynx and removed intact without mucosal injury. Following extraction, bronchoscopic examination demonstrated no residual endobronchial lesions or secretions, and post-procedural examination of the larynx showed no evidence of injury. The patient was admitted for overnight observation and remained clinically stable without procedure-related or respiratory complications. No additional imaging was performed, as there was no clinical indication following successful retrieval. He was discharged home the following day in good condition without any reported procedure-related complications.

## Discussion

This case illustrates three interrelated challenges that arise when foreign body aspiration occurs in a head and neck cancer survivor with radiation-induced trismus: the elevated baseline risk of aspiration, the anatomic constraints that limit conventional retrieval techniques, and the need to adapt procedural strategy to the patient’s specific airway anatomy.

Aspiration risk in this population

Dental foreign body aspiration in head and neck cancer survivors is uncommon but clinically important. Radiation-induced fibrosis impairs coordinated swallowing, sensory neuropathy diminishes protective laryngeal reflexes, xerostomia compromises bolus clearance, and accelerated dental deterioration increases the likelihood of dental interventions, each of which contributes to aspiration risk. In a multicenter cohort of 138 adults with tracheobronchial foreign body aspiration, iatrogenic events accounted for 37.0% of cases, while 21.0% of aspiration events occurred in dental clinics [1]. The long-term consequences may be substantial. Among 3,513 patients with head and neck cancer treated with concurrent chemoradiotherapy, the five-year cumulative incidence of aspiration pneumonia was 23.8% (95% CI, 22.3%-25.3%) [4].

Trismus as a constraint on retrieval

Radiation-induced trismus presents a unique procedural challenge by limiting the oral access required for conventional airway interventions. Rigid bronchoscopy remains the preferred technique for removal of large, sharp, or irregular tracheobronchial foreign bodies because it provides superior airway control, accommodates larger retrieval instruments, and facilitates management of airway bleeding or obstruction [5,7]. However, successful rigid bronchoscopy requires adequate interincisal opening to accommodate both the bronchoscope and a bite block, making it poorly suited for patients with severe trismus [6]. In our patient, a standard adult bite block could not be safely accommodated, and pediatric bite blocks were unavailable at our institution, precluding the rigid approach.

Flexible bronchoscopy is an established alternative for adult foreign body retrieval, with reported success rates approaching 90% [1,5]. Multiple retrieval devices, including forceps, baskets, snares, cryoprobes, and combinations of these techniques, have been described [2,8]. In patients with severe trismus, the transnasal route offers additional advantages by bypassing the restricted oral aperture while allowing the procedure to be performed under moderate sedation with preservation of spontaneous ventilation.

Rationale for the staged retrieval strategy

What distinguishes this case is not the choice of flexible over rigid bronchoscopy, but the subsequent decision to perform direct manual extraction from the oropharynx rather than attempting withdrawal of the bronchoscope, retrieval basket, and entrapped tooth through the severely restricted oral aperture. Two factors motivated this strategy. First, the irregular contour and sharp enamel edges of the molar created a substantial risk of laceration to the palate, nasopharynx, or pharyngeal mucosa during extraction through the limited oral opening. Second, withdrawal without a protective bite block increased the possibility of damage to the bronchoscope.

Patient repositioning to a slight Trendelenburg and left lateral decubitus position was intentionally performed before transfer of the foreign body from the basket to the forceps. Should the tooth have become dislodged during this step, gravity would favor movement toward the oropharynx, where it would be more readily accessible or expectorated, rather than allowing distal migration into the tracheobronchial tree. Previous reports involving trismus have focused primarily on airway management during intubation rather than bronchoscopic foreign body retrieval itself [6,9]. A recent report described dual tooth aspiration in a patient with post-radiation trismus managed with nasal fiberoptic bronchoscopy, with tracheostomy ultimately required for retrieval of one of the aspirated teeth [10]. In contrast, reports describing the specific staged sequence used in our case, transnasal flexible bronchoscopic capture, delivery into the oropharynx, and subsequent direct manual extraction, remain extremely limited.

Limitations

This report describes the successful management of a single patient and should not be interpreted as establishing a standard approach. The technique is best suited for carefully selected foreign bodies that can be securely captured with a retrieval basket but present an unacceptable risk during conventional oral withdrawal. Successful application requires experienced bronchoscopists, immediate anesthesia support, and access to surgical or otolaryngology backup should airway compromise or failed retrieval occur. Otolaryngology services were available at our institution if emergency surgical airway management became necessary.

## Conclusions

Foreign body aspiration should be recognized as a potential complication of dental procedures in patients with prior head and neck radiotherapy and severe trismus. When conventional oral or rigid bronchoscopy is not technically feasible because of severely restricted mouth opening, a staged approach using transnasal flexible bronchoscopy followed by direct oropharyngeal extraction may represent a feasible alternative in carefully selected patients. Based on this single case, individualized procedural planning, multidisciplinary collaboration, and adaptation of airway management strategies to the patient’s anatomy may help optimize outcomes in this challenging clinical setting.
